# Supplementary material for: Genetic Characterization of the Acidic and Neutral Glycosphingolipid Biosynthetic Pathways in Neurospora crassa
Source: Microorganisms. 2023 Aug 16;11(8):2093. doi: 10.3390/microorganisms11082093 (PMC10457978; doi:10.3390/microorganisms11082093)
Supplement: Supplementary file 1 [file microorganisms-11-02093-s001.zip › microorganisms-2555109-supplementary.pdf]

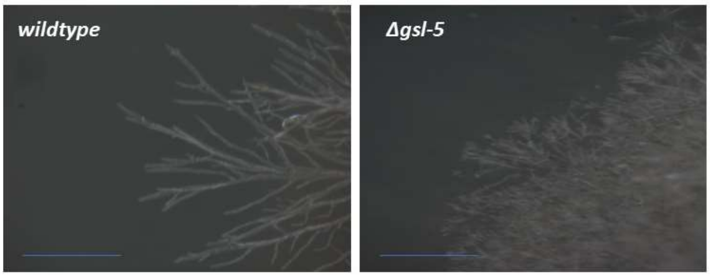

Figure S1. Morphology of the wildtype strain and the Δ*gsl-5* mutant strain. The wildtype and mutant isolate were inoculated on Vogel's 2% sucrose agar medium, and the growing edges of the colonies were viewed in a dissecting microscope. The edges of the growing colonies were photographed with a Canon Powershot A620 camera using overhead lighting.

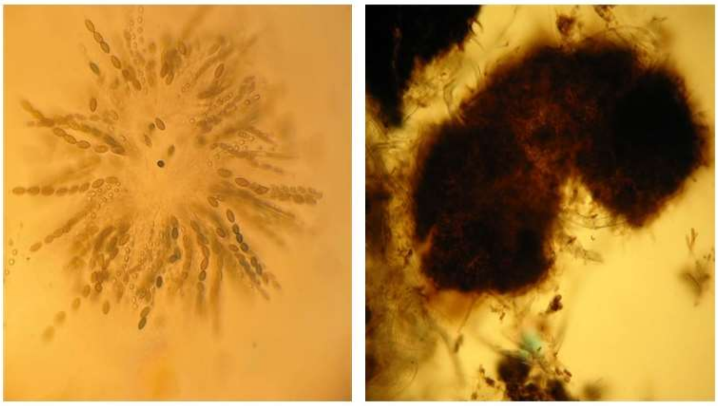

Figure S2. Ascospore development in wildtype and Δ*gsl-5* matings. Wildtype x wildtype and Δ*gsl-5* x Δ*gsl-5* matings were performed and the perithecia were allowed to develop for 18 days at 22ºC. Perithecia were collected and squashed to release ascospores. In the wildtype x wildtype mating, healthy melanized ascospores were released from the perithecia. The image shown is of a "rosette" of developing ascospores. The perithecia in the Δ*gsl-5* x Δ*gsl-5* matings were barren (no ascospores). The dark objects seen in the image of the mutant mating are broken perithecia. Note the absence of ascospores.
